# Fauna Europaea: Annelida – Hirudinea, incl. Acanthobdellea and Branchiobdellea

**DOI:** 10.3897/BDJ.2.e4015

**Published:** 2014-11-14

**Authors:** Alessandro Minelli, Boris Sket, Yde de Jong

**Affiliations:** † University of Padova, Padova, Italy; ‡ University of Ljubljana, Ljubljana, Slovenia; § University of Eastern Finland, Joensuu, Finland; | University of Amsterdam - Faculty of Science, Amsterdam, Netherlands

**Keywords:** Annelida, Hirudinea, Acanthobdellea, Branchiobdellea, leeches, Europe

## Abstract

*Fauna Europaea* provides a public web-service with an index of scientific names (including important synonyms) of all living European land and freshwater animals, their geographical distribution at country level (up to the Urals, excluding the Caucasus region), and some additional information. The *Fauna Europaea* project covers about 230,000 taxonomic names, including 130,000 accepted species and 14,000 accepted subspecies, which is much more than the originally projected number of 100,000 species. This represents a huge effort by more than 400 contributing specialists throughout Europe and is a unique (standard) reference suitable for many users in science, government, industry, nature conservation and education.

Hirudinea is a fairly small group of Annelida, with about 680 described species, most of which live in freshwater habitats, but several species are (sub)terrestrial or marine. In the Fauna Europaea database the taxon is represented by 87 species in 6 families. Two closely related groups, currently treated as distinct lineages within the Annelida, are the Acanthobdellea (2 species worldwide, of which 1 in Europe) and the Branchiobdellea (about 140 species worldwide, of which 10 in Europe). This paper includes a complete list of European taxa belonging to the Hirudinea, Acanthobdellea and Branchiobdellea. Recent research on a limited number of taxa suggests that our current appreciation of species diversity of Hirudinea in Europe is still provisional: on the one hand, cryptic, unrecognised taxa are expected to emerge; on the other, the status of some taxa currently treated as distinct species deserves revisiting.

## Introduction

The European Commission published the European Community Biodiversity Strategy, providing a framework for development of Community policies and instruments in order to comply with the Convention on Biological Diversity. This Strategy recognises the current incomplete state of knowledge at all levels concerning biodiversity, which is a constraint on the successful implementation of the Convention. Fauna Europaea contributes to this Strategy by supporting one of the main themes: to identify and catalogue the components of European biodiversity into a database in order to serve as a basic tool for science and conservation policies.

With regard to biodiversity in Europe, both science and policies depend on a knowledge of its components. The assessment of biodiversity, monitoring changes, sustainable exploitation of biodiversity, and much legislative work depend upon a validated overview of taxonomic biodiversity. Towards this end Fauna Europaea plays a major role, providing a web-based information infrastructure with an index of scientific names (including important synonyms; i.e. alternative names still in use, or at lead found in recent literature) of all living European land and freshwater animals, their geographical distribution at country level and some additional useful information. In this sense, the Fauna Europaea database provides a unique reference for many user-groups such as scientists, governments, industries, conservation communities and educational programs.

Fauna Europaea started in 2000 as an EC-FP5 four-year project, delivering its first release in 2004. After thirteen years of steady progress, in order to improve the dissemination of Fauna Europaea results and to increase the general awareness and acknowledgement of Fauna Europaea contributors, novel e-Publishing tools have been used to prepare data papers of all 58 major taxonomic groups (Jong et al. 2014).

### Data-papers & gap-analysis

In order to improve the dissemination and citation of Fauna Europaea and to increase the acknowledge of the Fauna Europaea contributors, a special Biodiversity Data Journal Series has been compiled using novel e-Publishing tools, called Contributions on Fauna Europaea, preparing data-papers of all major Fauna Europaea taxonomic groups. This work was initiated during the ViBRANT project and is further supported by the recently started EU BON project. This contribution represents the first publication of the Fauna Europaea Annelida–Hirudinea data sector as a BDJ data paper.

In the EU BON project (Hoffmann et al. 2014) also further steps will be made on implementing Fauna Europaea as a basic tool for biodiversity research and for taxonomic expertise evaluation and management in Europe, using Fauna Europaea as a standard reference for taxonomic Information. The Fauna Europaea data-papers will contribute to a quality assessement on biodiversity data by providing estimates on gaps in taxonomic information and knowledge (see Table 1).

## General description

### Purpose

Fauna Europaea is a database of the scientific names and distribution of all living, currently known European land and fresh-water animal species assembled by a large network of experts. An extended description of the Fauna Europaea project can be found in Jong et al. 2014. A summary is given in the sections below.

The Annelida–Hirudinea, together with their allies Acanthobdellea and Branchiobdellea, are one of the 58 Fauna Europaea major taxonomic groups, covering 98 species (Fig. 2). The data were acquired and checked by two specialists (Tables 1, 2).

### Additional information

The Hirudinea or leeches form a quite small group of macrophagous or blood-sucking annelids, including about 680 species (Sket and Trontelj 2008), probably derived from a lineage of freshwater oligochaetes. Their presence in the sea, and in terrestrial habitats, is secondary, following a primary radiation in freshwater habitats. About 15% of the ca. 680 species described thus far (Sket and Trontelj 2008), including many representatives of the Piscicolidae and all members of the Ozobranchidae, are marine; ca. 100 species (all members of the Americobdellidae, Cylicobdellidae, Haemadipsidae, Xerobdellidae, a few Hirudinidae and one member of the Salifidae) are terrestrial or semiterrestrial, all the remaining Hirudinea live in freshwater habitats. Three main feeding styles can be recognized among the leeches. The Rhynchobdellida, represented in the continental fauna of Europe by members of the Glossiphoniidae (Fig. 3) and the Piscicolidae, are provided with a stiff protrusible proboscis through which they pierce the skin of their preys (cold- and warm-blooded vertebrates, mollusks, insect larvae) and suck their body fluids or tissues; within the Arhynchobdellida, many representatives of the Hirudiniformes (in Europe, those of the family Hirudinidae) are provided with toothed muscular ‘mandibles’ used to produce superficial wounds in the skin of their vertebrate hosts, the blood of which they feed on, while those of other families (in Europe, Haemopidae and Xerobdellidae (Fig. 4), and also the Erpobdelliformes (only family Erpodbellidae native in Europe) are macrophagous and ingest whole preys, e.g. insect larvae, crustaceans, oligochaetes. Most leeches are temporary ectoparasites, their contact with their hosts being limited to the feeding phase, but many members of the Rhynchobdellida live more ore less permanently attached. All leeches are hermaphrodite. Large species may suck human blood. *Hirudo* spp. have been traditionally used in medicine.

The Annelida–Hirudinea database in Fauna Europaea includes also information on two smaller annelid groups, the Branchiobdellea and the Acanthobdellea, often treated in the past as members of the Hirudinea, in which case the true leeches (i.e., the Hirudinea of most current classifications) are often renamed Euhirudinea (e.g., Siddall et al. 2001). Today, the Hirudinea (in strict sense, corresponding to Euhirudinea of other classifications), Branchiobdellea and Acanthobdellea are treated by most authorities as distinct lineages within the clitellate annelids, alongside the taxa traditionally grouped as the Oligochaeta, Acanthobdellea and Branchiobdellea. All Branchiobdellea, ca. 140 species worldwide (Gelder 2010), are small (1–12 mm) freshwater worms that live on crustaceans, mainly crayfish. The Acanthobdellea, 2 species of cold riverine waters, are parasitic on fish, mainly salmon.

Molecular phylogenetic studies have heavily impacted on the internal classification of the Hirudinea (e.g., Borda and Siddall 2004) and more progress is expected from further studies. Detailed molecular systematics studies have shown gross errors in the traditional delineation of leech species, including the case of the European medicinal leech – a concept under which other species must be recognized besides *Hirudo medicinalis* Linnaeus, 1758 (Trontelj and Utevsky 2005). On the other hand, reproductive isolation seems not to be completed between lineages currently treated as distinct species, such as within the same group of species (S. Utevsky, pers. com.). Discrepancies between the results of morphological vs. molecular systematic analyses have also been noted in other groups, as in the endemic lineage of *Dina* species inhabiting Lake Ochrid (Trajanovski et al. 2010) and future reassessments are likely to increase the number of taxa worth recognition at the species level.

A revised delineation of genera and families is also suggested by a first molecular taxonomic study on Branchiobdellea (Williams et al. 2013).

## Project description

### Title

This BDJ data paper includes the taxonomic indexing efforts in Fauna Europaea on European Annelida–Hirudinea covering the first two versions of Fauna Europaea worked on between 2000 and 2013 (up to version 2.6).

### Personnel

The taxonomic framework of Fauna Europaea includes partner institutes, providing taxonomic expertise and information, and expert networks taking care of data collation.

Every taxonomic group is covered by at least one Group Coordinator responsible for the supervision and integrated input of taxonomic and distributional data for a particular group. The Fauna Europaea checklist would not have reached its current level of completion without the input from several groups of specialists. The formal responsibility of collating and delivering the data of relevant families rested with a number of Taxonomic Specialists Table 1. For Annelida–Hirudinea the Group Coordinator is Alessandro Minelli, who is also Taxonomic Specialist together with Boris Sket. Associate Specialist Serge Utevsky deserves credit for his important contributions at various levels, especially for Hirudinidae, and for distribution in Eastern European Table 2 countries.

A more detailed overview of the Fauna Europaea classification and expertise network for Annelida–Hirudinea can be found here: http://www.faunaeur.org/experts.php?id=80.

Data management tasks are taken care of by the Fauna Europaea project bureau. During the project phase (until 2004) a network of principal partners took care of diverse management tasks: Zoological Museum Amsterdam (general management & system development), Zoological Museum of Copenhagen (data collation), National Museum of Natural History in Paris (data validation) and Museum and Institute of Zoology in Warsaw (NAS extension). Since the formal project ending (from 2004, till 2014) all tasks have been undertaken by the Zoological Museum Amsterdam.

### Study area description

The area study covers the European mainland (Western Palearctic), including the Macaronesian islands, excluding the Caucasus, Turkey, Arabian Peninsula and Northern Africa (see: Geographic coverage).

### Design description

Standards. Group coordinators and taxonomic specialists have to deliver the (sub)species names according to strict standards. The names provided by Fauna Europaea are scientific names. The taxonomic scope includes issues like, (1) the definition of criteria used to identify the accepted species-group taxa, (2) the hierarchy (classification scheme) for the accommodation of the all accepted species, (3) relevant synonyms, and (4) the correct nomenclature. The Fauna Europaea 'Guidelines for Group Coordinators and Taxonomic Specialists', include the standards, protocols, scope, and limits that provide the instructions for the more than 400 specialists contributing to the project, strictly following the provisions of the current edition of the International Code of Zoological Nomenclature.

Data management. The data records could either be entered offline into a preformatted MS-Excel worksheet or directly into the Fauna Europaea transaction database using an online browser interface (see Fig. 5). Since 2013 the data servers are hosted at the Museum für Naturkunde in Berlin.

Data set. The Fauna Europaea basic data set consists of: accepted (sub)species names (including authorship), synonym names (including authorship), a taxonomic hierarchy/classification, misapplied names (including misspellings and alternative taxonomic views), homonym annotations, expert details, European distribution (at country level), Global distribution (only for European species), taxonomic reference (optional), occurrence reference (optional).

### Funding

Fauna Europaea was funded by the European Commission under various framework programs (see Acknowledgement).

## Sampling methods

### Study extent

See spatial coverage and geographic coverage descriptions.

### Sampling description

Fauna Europaea data have been assembled by principal taxonomic experts, based on their individual expertise, including literature sources, collection research, and field observations. In total no less than 476 experts contributed taxonomic and/or faunistic information to Fauna Europaea. The vast majority of the experts are from Europe (including EU non-member states). As a unique feature, Fauna Europaea funds were set aside for rewarding/compensating for the work of taxonomic specialists and group coordinators.

To facilitate data transfer and data import, sophisticated on-line (web interfaces) and off-line (spreadsheets) data-entry routines were built, integrated within an underlying central Fauna Europaea transaction database (see Fig. 5).

A first release of the Fauna Europaea index via the web-portal has been presented on the 27^th^ of September 2004, the most recent release (version 2.6.2) was launched on the 29th August 2013. An overview of Fauna Europaea releases can be found here: http://www.faunaeur.org/about_fauna_versions.php.

### Quality control

Fauna Europaea data are unique in the sense that they are fully expert-based. Selecting leading experts for all groups assured the systematic reliability and consistency of the Fauna Europaea data.

Furthermore, all Fauna Europaea data sets are intensively reviewed at regional and thematic validation meetings, at review sessions on taxonomic symposia (for some groups), by Fauna Europaea Focal Points (during the FaEu-NAS and PESI projects) and by various end-users sending annotations using the web form at the web-portal. Additional validation on gaps and correct spelling was effected at the validation office in Paris.

In conclusion, in general we expect to get taxonomic data for 99.3% of the known European fauna. The faunistic coverage is not quite as good, but is nevertheless 90–95% of the total fauna. For the Annelida–Hirudinea (this paper) the taxonomic coverage is 100% (see Table 1), but the distribution by country is still incomplete, especially for the Branchiobdellea.

Checks on technical and logical correctness of the data have been implemented in the data entry tools, including around 50 "Taxonomic Integrity Rules". This validation tool proved to be of huge value for both the experts and project management, and contributed significantly to preparation of a remarkably clean and consistent data set. This thorough reviewing makes Fauna Europaea the most scrutinised data sets in its domain.

### Step description

By evaluating team structure and life cycle procedures (data-entry, validation, updating, etc.), clear definitions of roles of users and user-groups, according to the taxonomic framework were established, including ownership and read and writes privileges, and their changes during the project life-cycle. In addition, guidelines on common data exchange formats and codes have been issued (see also the 'Guidelines for Experts' document).

## Geographic coverage

### Description

**Figure 1.**
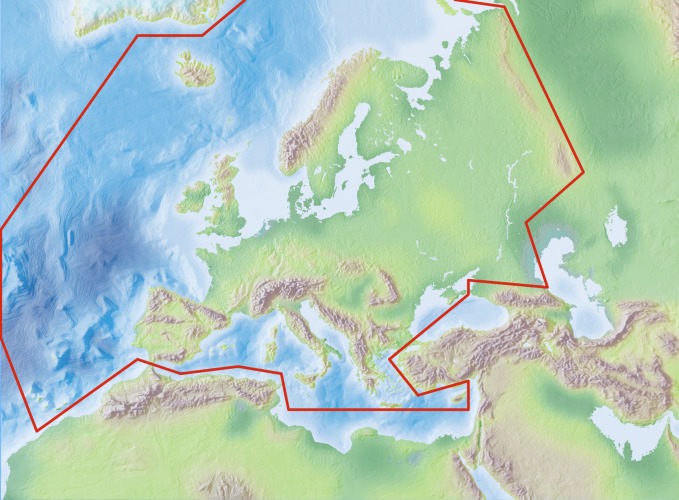
Fauna Europaea geographic coverage ('minimal Europe').

Species and subspecies distributions in Fauna Europaea are registered at least a country level, i.e. for political countries. For this purpose the FaEu geographical system basically follows the TDWG standards. The covered area includes the European mainland (Western Palearctic), plus the Macaronesian islands (excl. Cape Verde Islands), Cyprus, Franz Josef Land and Novaya Zemlya. Western Kazakhstan and the Caucasus are excluded (see Fig. 1).

The focus is on species (or subspecies) of European animals of terrestrial and freshwater environments. Species in brackish waters, occupying the marine/freshwater or marine/terrestrial transition zones, are generally excluded.

### Coordinates

Mediterranean (N 35°) and Arctic Islands (N 82°) Latitude; Atlantic Ocean (Mid-Atlantic Ridge) (W 30°) and Ural (E 60°) Longitude.

## Taxonomic coverage

### Description

The Fauna Europaea database contains the scientific names of all living European land and freshwater animal species, including numerous infra-groups and synonyms. More details about the conceptual background of Fauna Europaea and standards followed are described above and in the project description paper(s).

**Figure 2.**
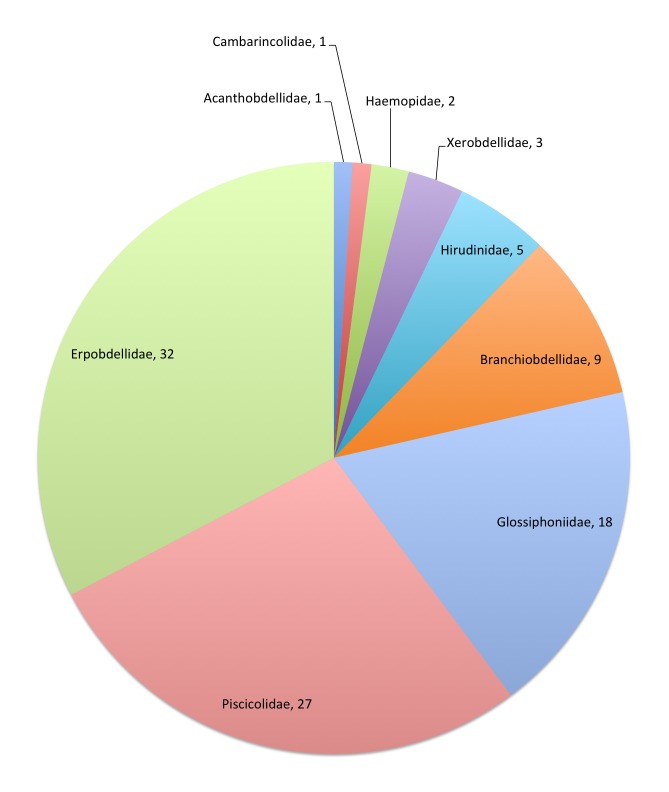
FaEu Annelida–Hirudinea species per family. See Table 1 for family statistics.

**Figure 3.**
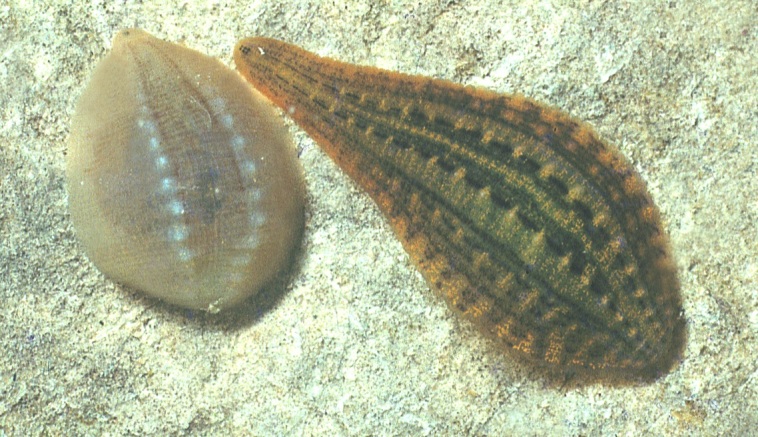
The common snail leech *Glossiphonia complanata complanata* (right) and the lake Ohrid endemic *Glossiphonia pulchella*.

**Figure 4.**
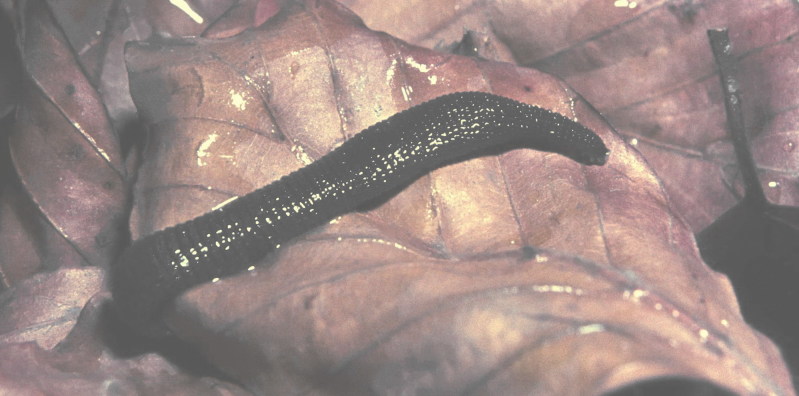
The European terrestrial leech *Xerobdella lecomtei*.

**Figure 5.**
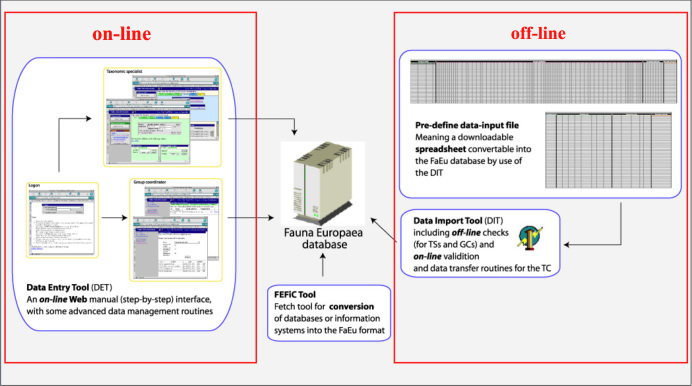
Fauna Europaea on-line (browser interfaces) and off-line (spreadsheets) data entry tools.

This data paper covers the Annelida–Hirudinea content of Fauna Europaea, including 9 families, 98 species, 9 subspecies and 42 (sub)species synonyms (see Fig. 2).

All species described to date are included in the current version of the data base. We may expect a future increase of species numbers for Erpobdellidae (ca 10 or even more species), and perhaps one or two species in other families, e.g. Glossiphoniidae and Piscicolidae (see Table 1). A reliable assessment of the expected number of species is not feasible at present for the Branchiobdellidae.

### Taxa included

**Table:** 

Rank	Scientific Name	Common Name
subkingdom	Eumetazoa	
kingdom	Animalia	
phylum	Annelida	
class	Acanthobdellea	
class	Branchiobdellea	
class	Hirudinea	
order	Acanthobdellida	
order	Arhynchobdellida	
order	Branchiobdellida	
order	Rhynchobdellida	
suborder	Erpobdelliformes	
suborder	Hirudiniformes	
family	Acanthobdellidae	
family	Branchiobdellidae	
family	Cambarincolidae	
family	Erpobdellidae	
family	Glossiphoniidae	
family	Haemopidae	
family	Hirudinidae	
family	Piscicolidae	
family	Xerobdellidae	
family	Haemadipsidae	
genus	*Acanthobdella* Grube 1850	
genus	*Branchiobdella* Odier 1823	
genus	*Xironogiton* Ellis 1920	
genus	*Cambarincola* Ellis 1912	
genus	*Archaeobdella* Grimm 1876	
genus	*Barbronia* R. Blanchard 1897	
genus	*Dina* R. Blanchard 1892	
genus	*Erpobdella* Blainville 1818	
genus	*Fadejewobdella* Lukin 1962	
genus	*Trocheta* Dutrochet 1817	
genus	*Alboglossiphonia* Lukin 1976	
genus	*Batracobdella* Viguier 1879	
genus	*Batracobdelloides* Oosthuizen 1984	
genus	*Glossiphonia* Johnson 1816	
genus	*Helobdella* R. Blanchard 1896	
genus	*Hemiclepsis* Vejdovsky 1884	
genus	*Placobdella* R. Blanchard 1893	
genus	*Theromyzon* Philippi 1867	
genus	*Haemopis* Savigny 1822	
genus	*Hirudo* Linnaeus 1758	
genus	*Limnatis* Moquin-Tandon 1826	
genus	*Calliobdella* Van Beneden & Hesse 1863	
genus	*Caspiobdella* Epshtein 1966	
genus	*Croatobranchus* Kozarčanin 1995	
genus	*Cystobranchus* Diesing 1859	
genus	*Italobdella* Bielecki 1993	
genus	*Pawlowskiella* Bielecki 1997	
genus	*Piscicola* Blainville 1818	
genus	*Xerobdella* von Frauenfeld 1868	
species	*Dina absoloni* Johansson 1913	
species	*Batracobdella algira* (Moquin-Tandon 1846)	
species	*Piscicola annae* Bielecki 1997	
species	*Xerobdella anulata* Autrum 1958	
species	*Dina apathyi* Gedroyc 1916	
species	*Branchiobdella astaci* Odier 1823	
species	*Branchiobdella balcanica* Moszynski 1937	
species	*Piscicola borowieci* Bielecki 1997	
species	*Piscicola brylinskae* Bielecki 2001	
species	*Trocheta bykowskii* Gedroyc 1913	
species	*Haemopis caeca* Manoleli, Klemm & Sarbu 1998	
species	*Caspiobdella caspica* (Selensky 1915)	
species	*Italobdella ciosi* Bielecki 1993	
species	*Glossiphonia complanata* (Linnaeus 1758)	
species	*Glossiphonia concolor* (Apáthy 1888)	
species	*Placobdella costata* (Fr. Müller 1846)	
species	*Trocheta cylindrica* Örley 1886	
species	*Trocheta dalmatina* Sket 1968	
species	*Piscicola elishebae* Bielecki 1997	
species	*Italobdella epshteini* Bielecki 1997	
species	*Archaeobdella esmonti* Grimm 1876	
species	*Dina eturpshem* Sket 1989	
species	*Caspiobdella fadejewi* Epshtein 1961	
species	*Trocheta falkneri* Nesemann & Neubert 1996	
species	*Cystobranchus fasciatus* (Kollar 1842)	
species	*Piscicola geometra* (Linnaeus 1758)	
species	*Piscicola hadzii* Sket 1985	
species	*Piscicola haranti* Jarry 1960	
species	*Trocheta haskonis* Grosser 2000	
species	*Alboglossiphonia heteroclita* (Linnaeus 1761)	
species	*Branchiobdella hexodonta* Grube 1888	
species	*Alboglossiphonia hyalina* (O.F. Müller 1774)	
species	*Xironogiton instabilis* (Moore 1894)	
species	*Branchiobdella italica* Canegallo 1928	
species	*Piscicola jarai* Bielecki 1997	
species	*Branchiobdella kozarovi* Subchev 1978	
species	*Dina krasensis* (Sket 1968)	
species	*Dina krilata* Sket 1989	
species	*Piscicola kusznierzi* Bielecki 1997	
species	*Dina kuzmani* Šapkarev 1990	
species	*Dina latestriata* Neubert & Nesemann 1995	
species	*Xerobdella lecomtei* von Frauenfeld 1868	
species	*Dina lepinja* Sket & Šapkarev 1986	
species	*Dina lineata* (O.F. Müller 1774)	
species	*Dina lyhnida* Šapkarev 1990	
species	*Theromyzon maculosum* (Rathke 1862)	
species	*Calliobdella mammillata* (Malm 1863)	
species	*Piscicola margaritae* Bielecki 1997	
species	*Hemiclepsis marginata* (O.F. Müller 1774)	
species	*Hirudo medicinalis* Linnaeus 1758	
species	*Cambarincola mesochoreus* Hoffman 1963	
species	*Erpobdella monostriata* (Lindenfeld & Pietruszynski 1890)	
species	*Batracobdelloides moogi* Nesemann & Csanyi 1995	
species	*Glossiphonia nebulosa* Kalbe 1964	
species	*Piscicola niewiadomskae* Bielecki 1997	
species	*Erpobdella nigricollis* (Brandes 1900)	
species	*Limnatis nilotica* (Savigny 1822)	
species	*Dina ochridana* Sket 1968	
species	*Erpobdella octoculata* (Linnaeus 1758)	
species	*Glossiphonia paludosa* (Carena 1824)	
species	*Branchiobdella papillosa* Hutter & Nesemann 2001	
species	*Alboglossiphonia papillosa* (Braun 1805)	
species	*Branchiobdella parasita* (Braun 1805)	
species	*Cystobranchus pawlowskii* Sket 1968	
species	*Acanthobdella peledina* Grube 1850	
species	*Branchiobdella pentodonta* Whitman 1882	
species	*Piscicola pojmanskae* Bielecki 1994	
species	*Piscicola pomorskii* Bielecki 1997	
species	*Xerobdella praealpina* Minelli 1971	
species	*Dina profunda* Šapkarev 1990	
species	*Trocheta pseudodina* Nesemann 1990	
species	*Glossiphonia pulchella* Sket 1968	
species	*Dina punctata* Johansson 1927	
species	*Calliobdella punctata* Van Beneden & Hesse 1863	
species	*Fadejewobdella quinqueannulata* (Lukin 1929)	
species	*Cystobranchus respirans* (Troschel 1850)	
species	*Trocheta riparia* Nesemann 1993	
species	*Haemopis sanguisuga* (Linnaeus 1758)	
species	*Glossiphonia slovaca* (Kosel 1973)	
species	*Helobdella stagnalis* (Linnaeus 1758)	
species	*Pawlowskiella stenosa* Bielecki 1997	
species	*Dina stschegolewi* (Lukin & Epshtein 1960)	
species	*Trocheta subviridis* Dutrochet 1817	
species	*Dina svilesta* Sket 1989	
species	*Theromyzon tessulatum* (O.F. Müller 1774)	
species	*Erpobdella testacea* (Savigny 1820)	
species	*Helobdella triserialis* (E. Blanchard 1849)	
species	*Hirudo troctina* Johnson 1816	
species	*Caspiobdella tuberculata* Epshtein 1966	
species	*Hirudo verbana* Carena 1820	
species	*Glossiphonia verrucata* (Fr. Müller 1844)	
species	*Erpobdella vilnensis* (Liskiewicz 1925)	
species	*Caspiobdella volgensis* (Zykoff 1903)	
species	*Barbronia weberi* (R. Blanchard 1897)	
species	*Piscicola wiktori* Bielecki 1997	
species	*Piscicola witkowskii* Bielecki 1997	
subspecies	*Branchiobdella balcanica balcanica* Moszynski 1937	
subspecies	*Branchiobdella balcanica sketi* Karaman 1970	
subspecies	*Glossiphonia complanata complanata* (Linnaeus 1758)	
subspecies	*Glossiphonia complanata maculosa* Sket 1968	
subspecies	*Dina lineata concolor* (Annandale 1913)	
subspecies	*Dina lineata dinarica* Sket 1968	
subspecies	*Dina lineata lacustris* Sket 1968	
subspecies	*Dina lineata lineata* (O.F. Müller 1774)	
subspecies	*Dina lineata montana* Sket 1968	
species	*Croatobranchus mestrovi* Kozarčanin 1995	

## Temporal coverage

**Living time period:** Currently living.

### Notes

Currently living animals in stable populations, largely excluding (1) rare/irregular immigrants, intruder or invader species, (2) accidental or deliberate releases of exotic (pet) species, (3) domesticated animals, (4) foreign species imported and released for bio-control or (5) foreign species largely confined to hothouses.

## Usage rights

### Use license

Open Data Commons Attribution License

### IP rights notes

Fauna Europaea data are licensed under CC BY SA version 4.0. The property rights of experts over their data is covered under the SMEBD conditions. For more copyrights and citation details see: http://www.faunaeur.org/copyright.php.

## Data resources

### Data package title

Fauna Europaea - Annelida - Hirudinea

### Resource link

http://www.faunaeur.org/Data_papers/FaEu_Annelida-Hirudinea_2.6.2.zip

### Alternative identifiers

http://www.faunaeur.org/full_results.php?id=11261

### Number of data sets

2

### Data set 1.

#### Data set name

Fauna Europaea - Annelida - Hirudinea version 2.6.2 - species

#### Data format

CSV

#### Number of columns

25

#### Character set

UTF-8

#### Download URL

http://www.faunaeur.org/Data_papers/FaEu_Annelida-Hirudinea_2.6.2.zip

#### Description

**Data set 1.** 

Column label	Column description
datasetName	The name identifying the data set from which the record was derived (http://rs.tdwg.org/dwc/terms/datasetName).
version	Release version of data set.
versionIssued	Issue data of data set version.
rights	Information about rights held in and over the resource (http://purl.org/dc/terms/rights).
rightsHolder	A person or organization owning or managing rights over the resource (http://purl.org/dc/terms/rightsHolder).
accessRights	Information about who can access the resource or an indication of its security status (http://purl.org/dc/terms/accessRights).
taxonID	An identifier for the set of taxon information (http://rs.tdwg.org/dwc/terms/taxonID)
parentNameUsageID	An identifier for the name usage of the direct parent taxon (in a classification) of the most specific element of the scientificName (http://rs.tdwg.org/dwc/terms/parentNameUsageID).
scientificName	The full scientific name, with authorship and date information if known (http://rs.tdwg.org/dwc/terms/scientificName).
acceptedNameUsage	The full name, with authorship and date information if known, of the currently valid (zoological) taxon (http://rs.tdwg.org/dwc/terms/acceptedNameUsage).
originalNameUsage	The original combination (genus and species group names), as firstly established under the rules of the associated nomenclaturalCode (http://rs.tdwg.org/dwc/terms/originalNameUsage).
family	The full scientific name of the family in which the taxon is classified (http://rs.tdwg.org/dwc/terms/family).
familyNameId	An identifier for the family name.
genus	The full scientific name of the genus in which the taxon is classified (http://rs.tdwg.org/dwc/terms/genus).
subgenus	The full scientific name of the subgenus in which the taxon is classified. Values include the genus to avoid homonym confusion (http://rs.tdwg.org/dwc/terms/subgenus).
specificEpithet	The name of the first or species epithet of the scientificName (http://rs.tdwg.org/dwc/terms/specificEpithet).
infraspecificEpithet	The name of the lowest or terminal infraspecific epithet of the scientificName, excluding any rank designation (http://rs.tdwg.org/dwc/terms/infraspecificEpithet).
taxonRank	The taxonomic rank of the most specific name in the scientificName (http://rs.tdwg.org/dwc/terms/infraspecificEpithet).
scientificNameAuthorship	The authorship information for the scientificName formatted according to the conventions of the applicable nomenclaturalCode (http://rs.tdwg.org/dwc/terms/scientificNameAuthorship).
authorName	Author name information
namePublishedInYear	The four-digit year in which the scientificName was published (http://rs.tdwg.org/dwc/terms/namePublishedInYear).
Brackets	Annotation if authorship should be put between parentheses.
nomenclaturalCode	The nomenclatural code under which the scientificName is constructed (http://rs.tdwg.org/dwc/terms/nomenclaturalCode).
taxonomicStatus	The status of the use of the scientificName as a label for a taxon (http://rs.tdwg.org/dwc/terms/taxonomicStatus).
resourceDescription	An account of this resource, including a DOI of this data-paper (http://purl.org/dc/terms/description)

### Data set 2.

#### Data set name

Fauna Europaea - Annelida - Hirudinea version 2.6.2 - hierarchy

#### Data format

CSV

#### Number of columns

12

#### Character set

UTF-8

#### Download URL

http://www.faunaeur.org/Data_papers/FaEu_Annelida-Hirudinea_2.6.2.zip

#### Description

**Data set 2.** 

Column label	Column description
datasetName	The name identifying the data set from which the record was derived (http://rs.tdwg.org/dwc/terms/datasetName).
version	Release version of data set.
versionIssued	Issue data of data set version.
rights	Information about rights held in and over the resource (http://purl.org/dc/terms/rights).
rightsHolder	A person or organization owning or managing rights over the resource (http://purl.org/dc/terms/rightsHolder).
accessRights	Information about who can access the resource or an indication of its security status (http://purl.org/dc/terms/accessRights).
taxonName	The full scientific name of the higher-level taxon
scientificNameAuthorship	The authorship information for the scientificName formatted according to the conventions of the applicable nomenclaturalCode (http://rs.tdwg.org/dwc/terms/scientificNameAuthorship).
taxonRank	The taxonomic rank of the most specific name in the scientificName (http://rs.tdwg.org/dwc/terms/infraspecificEpithet).
taxonID	An identifier for the set of taxon information (http://rs.tdwg.org/dwc/terms/taxonID)
parentNameUsageID	An identifier for the name usage of the direct parent taxon (in a classification) of the most specific element of the scientificName (http://rs.tdwg.org/dwc/terms/parentNameUsageID).
resourceDescription	An account of this resource, including a DOI of this data-paper (http://purl.org/dc/terms/description)

## Figures and Tables

**Table 1.** Responsible specialists per family in Annelida–Hirudinea.

FAMILY	NUMBER OF SPECIES IN FAEU (in brackets, estimated actual number of existing species)	SPECIALIST(S)
Acanthobdellidae	1 (1)	Alessandro Minelli, Boris Sket
Branchiobdellidae	9 (9)	Alessandro Minelli, Boris Sket
Cambarincolidae	1 (1)	Alessandro Minelli, Boris Sket
Erpobdellidae	32 (~42)	Alessandro Minelli, Boris Sket
Glossiphoniidae	18 (~20)	Alessandro Minelli, Boris Sket
Haemopidae	2 (2)	Alessandro Minelli, Boris Sket
Hirudinidae	5 (5)	Alessandro Minelli, Boris Sket
Piscicolidae	27 (~29)	Alessandro Minelli, Boris Sket
Xerobdellidae	3 (3)	Alessandro Minelli, Boris Sket

**Table 2.** Responsible associated specialists in Annelida–Hirudinea.

GROUP or AREA	SPECIALIST(S)
Hirudinidae	Serge Utevsky
Eastern European countries	Serge Utevsky
